# Aberrant T cell responses in the bone marrow microenvironment of patients with poor graft function after allogeneic hematopoietic stem cell transplantation

**DOI:** 10.1186/s12967-017-1159-y

**Published:** 2017-03-14

**Authors:** Yuan Kong, Yu-Tong Wang, Xie-Na Cao, Yang Song, Yu-Hong Chen, Yu-Qian Sun, Yu Wang, Xiao-Hui Zhang, Lan-Ping Xu, Xiao-Jun Huang

**Affiliations:** 1 Peking University People’s Hospital, Peking University Institute of Hematology, Beijing Key Laboratory of Hematopoietic Stem Cell Transplantation, Collaborative Innovation Center of Hematology, Peking University, Beijing, 100044 China; 2 Peking-Tsinghua Center for Life Sciences, Academy for Advanced Interdisciplinary Studies, Peking University, Beijing, 100871 China

**Keywords:** Poor graft function, Bone marrow immune microenvironment, Regulatory T cells, Th17 cells, Allogeneic hematopoietic stem cell transplantation

## Abstract

**Background:**

Poor graft function (PGF) is a life-threatening complication after allogeneic hematopoietic stem cell transplantation (allo-HSCT). Nevertheless, whether abnormalities of T cell subsets in the bone marrow (BM) immune microenvironment, including Th17, Tc17, Th1, Tc1, Th2, Tc2 cells and regulatory T cells (Tregs), are involved in the pathogenesis of PGF remains unclear.

**Methods:**

This prospective nested case–control study enrolled 20 patients with PGF, 40 matched patients with good graft function (GGF) after allo-HSCT, and 20 healthy donors (HD). Th17, Tc17, Th1, Tc1, Th2, Tc2 cells, Tregs and their subsets were analyzed by flow cytometry.

**Results:**

A significantly higher proportion of stimulated CD4^+^ and CD8^+^ T cells that produced IL-17 (Th17 and Tc17) was found in the BM of PGF patients than in the BM of GGF patients and HD, whereas the percentages of Tregs in PGF patients were comparable to those in GGF patients and HD, resulting in a dramatically elevated ratio of Th17 cells/Tregs in the BM of PGF patients relative to those in GGF patients. Moreover, both CD4^+^ and CD8^+^ T cells were polarized towards a type 1 immune response in the BM of PGF patients.

**Conclusions:**

The present study revealed that aberrant T cell responses in the BM immune microenvironment may be involved in the pathogenesis of PGF after allo-HSCT. These findings will facilitate the optimization of immune regulation strategies and improve the outcome of PGF patients post-allotransplant.

**Electronic supplementary material:**

The online version of this article (doi:10.1186/s12967-017-1159-y) contains supplementary material, which is available to authorized users.

## Background

Poor graft function (PGF) remains a life-threatening complication following allogeneic hematopoietic stem cell transplantation (allo-HSCT), and the underlying mechanisms have not yet been elucidated [1–3]. Considerable evidence from murine studies has demonstrated that effective hematopoiesis depends on the specific bone marrow (BM) microenvironment, where hematopoietic stem cells (HSCs) reside [4–6]. BM endosteal cells, endothelial progenitor cells (EPCs), and perivascular cells have been regarded as the preferential elements that support hematopoiesis in the BM microenvironment [7, 8]. In this regard, we recently reported that even if the CD34^+^ BM cells from transplanted donors are functionally normal pre-transplant, reactive oxygen species-induced apoptosis may contribute to the exhaustion of CD34^+^ BM cells in patients with PGF following allo-HSCT [9]. Additionally, PGF patients demonstrate defective BM EPCs, endosteal cells, and perivascular cells in the BM microenvironment [10, 11]. Moreover, atorvastatin may improve the ability of impaired BM EPCs to support HSCs in PGF patients, although these findings were not statistically significant, as determined by colony-forming unit plating efficiency in vitro [12]. Together, these data appear to suggest that the impaired BM microenvironment may hamper the hematopoietic reconstitution of successfully engrafted donor HSCs, ultimately leading to the occurrence of PGF post-allo-HSCT.

In addition to the aforementioned elements of the BM microenvironment, various mature immune cell types, including T cells, B cells, dendritic cells, and macrophages, constitute the BM immune microenvironment and regulate the process of hematopoiesis [13, 14]. Clinical data and murine studies have shown that aberrant T cell responses in the BM microenvironment may exacerbate cytopenia and the dysfunction of HSCs and EPCs [15, 16]. Although the enrolled numbers of patients with PGF (N = 10) or good graft function (GGF) (N = 20) were limited, our pilot study found that both CD4^+^ and CD8^+^ T cells were polarized towards a type 1 immune response in the BM microenvironment of PGF patients compared to those in matched GGF patients [17]. However, the pattern of other T cell subsets in the BM immune microenvironment of PGF patients remains to be explored.

In addition to Th1 and Th2 cells, Th17 cells are an independent helper T cell lineage that is characterized by the production of interleukin (IL)-17 [18]. It has been determined that Th17 cells play a role in inflammation and autoimmune disease [19, 20]. CD4^+^CD25^+^CD127^−/low^ regulatory T cells (Tregs), which express the forkhead transcription factor Foxp3, are considered a group of suppressor T cells that are key players in the regulation of immune responses [21]. Th17 cells and Tregs are reciprocally related to each other. For example, TGF-β alone induces Treg differentiation, whereas Th17 differentiation is induced by TGF-β in combination with IL-6 and IL-21 [22]. The balance between Th17 cells and Tregs may shift towards IL-17-dominated pro-inflammatory responses during infection, autoimmune disease and graft-versus-host disease (GvHD) [23–26]. Th17 cells and Tregs also exist in the BM immune microenvironment and participate in the regulation of hematopoiesis [15, 16, 27]. However, less is known about Th17 cells and Tregs in the BM immune microenvironment of PGF patients.

Therefore, a prospective nested case–control study was conducted to evaluate whether the Th17, Tc17, Th1, Tc1, Th2, Tc2 cells, Tregs and their subsets in the BM immune microenvironment in allo-HSCT patients with PGF differ from those in patients with GGF or in healthy donors (HD). The aim of the current study was to provide new insights into the pathogenesis underlying PGF after allo-HSCT.

## Methods

### Patients and healthy controls

Twenty patients who had developed PGF after allo-HSCT were enrolled in this prospective nested case–control study. These cases were identified from patients who underwent allo-HSCT for hematological diseases between February 1, 2015 and January 31, 2016 at Peking University Institute of Hematology and were enrolled according to the following criteria: age at HSCT (±1 years), underlying disease, pre-HSCT cycles of chemotherapy (±1 cycle), and disease status at HSCT (“risk-set sampling”) [28]. Two matched GGF patients (n = 40) for each PGF case were randomly selected from the same cohort at the time PGF occurred. Among them, 10 patients with PGF and 20 matched GGF patients had been partially reported [17]. BM samples from 12 male and 8 female HD were used as healthy controls. The age of the HD ranged from 19 to 51 years (median: 39 years). This study was approved by the Ethics Committee of Peking University People’s Hospital. Informed consent was obtained from all patients and donors before entry into the study in accordance with the Declaration of Helsinki.

### Transplantation protocols

Donor selection, HLA typing, graft harvesting, conditioning therapy and GvHD prophylaxis were performed as previously reported [10, 29–32]. The subjects were screened for cytomegalovirus (CMV) infection by serology. Real-time quantitative PCR was used to detect CMV reactivation twice a week in blood samples. CMV infection was treated with ganciclovir or foscarnet as described [33]. After allo-HSCT, recombinant human granulocyte colony-stimulating factor (rhG-CSF) (5 μg/kg/day) was administered to the recipients of HLA-mismatched related transplants from day +6 until the neutrophil level was >0.5 × 10^9^/L for 3 consecutive days. rhG-CSF was not administered to recipients of HLA-identical sibling transplants, except in cases where neutrophil levels were <0.5 × 10^9^/L until day +21.

### Definition of good/poor graft function

Transplant recipients had to have complete donor hematological chimerism with no residual or recurrent leukemia. Good graft function [9–12, 17, 34] was characterized by an absolute neutrophil cell (ANC) count >0.5 × 10^9^/L for 3 consecutive days, platelet (PLT) count >20 × 10^9^/L for 7 consecutive days, and hemoglobin (Hb) level >70 g/L without transfusion support beyond day +28 post-HSCT. Poor graft function [9–12, 17] was defined as a hypo- or aplastic BM with 2 or 3 of the following characteristics: (1) ANC ≤ 0.5 × 10^9^/L; (2) PLT ≤ 20 × 10^9^/L; and/or (3) hemoglobin concentration ≤70 g/L for at least 3 consecutive days after day +28 post-HSCT or in accordance with platelet and/or red blood cell (RBC) transfusion and/or G-CSF support requirement. All RBC and PLT transfused to the patients post-HSCT were gamma irradiated. Patients with evidence of severe GvHD including III-IV acute GvHD and severe chronic GvHD or hematologic relapse after allo-HSCT were excluded.

Chimerism analyses were conducted using DNA fingerprinting for short tandem repeats (STRs) in blood samples and/or chromosome fluorescent in situ hybridization of bone marrow samples. Complete donor chimerism was defined as the detection of no recipient hematopoietic or lymphoid cells (sensitivity >0.1% recipient signals) [32].

### Surface immunophenotype analysis of T cell subsets

Bone marrow mononuclear cells (BMMNCs) were separated using Lymphocyte Separation Medium (HaoYang, Tianjin, China). Lymphocyte subsets were quantified by flow cytometry using the following directly conjugated mouse anti-human monoclonal antibodies: V500-conjugated anti-CD3, PerCP-conjugated anti-CD4, APC-conjugated anti-CD45RA, and PE-conjugated anti-CCR7 (BD Biosciences, San Jose, CA). After incubation, RBCs were lysed, and white blood cells (WBCs) were fixed with a lysing solution (BD Biosciences). As previously reported [17, 25, 35], effector T cells, naïve T cells, effector memory T cells, and central memory T cells were identified as CD45RA^+^CCR7^−^, CD45RA^+^CCR7^+^, CD45RA^−^CCR7^−^, and CD45RA^−^CCR7^+^, respectively. Multi-parameter flow cytometric analyses were performed using a BD LSRFortessa (Becton–Dickinson). Data were analyzed using BD Diva software (Becton–Dickinson).

### Intracellular cytokine staining

**Fig. 1:**
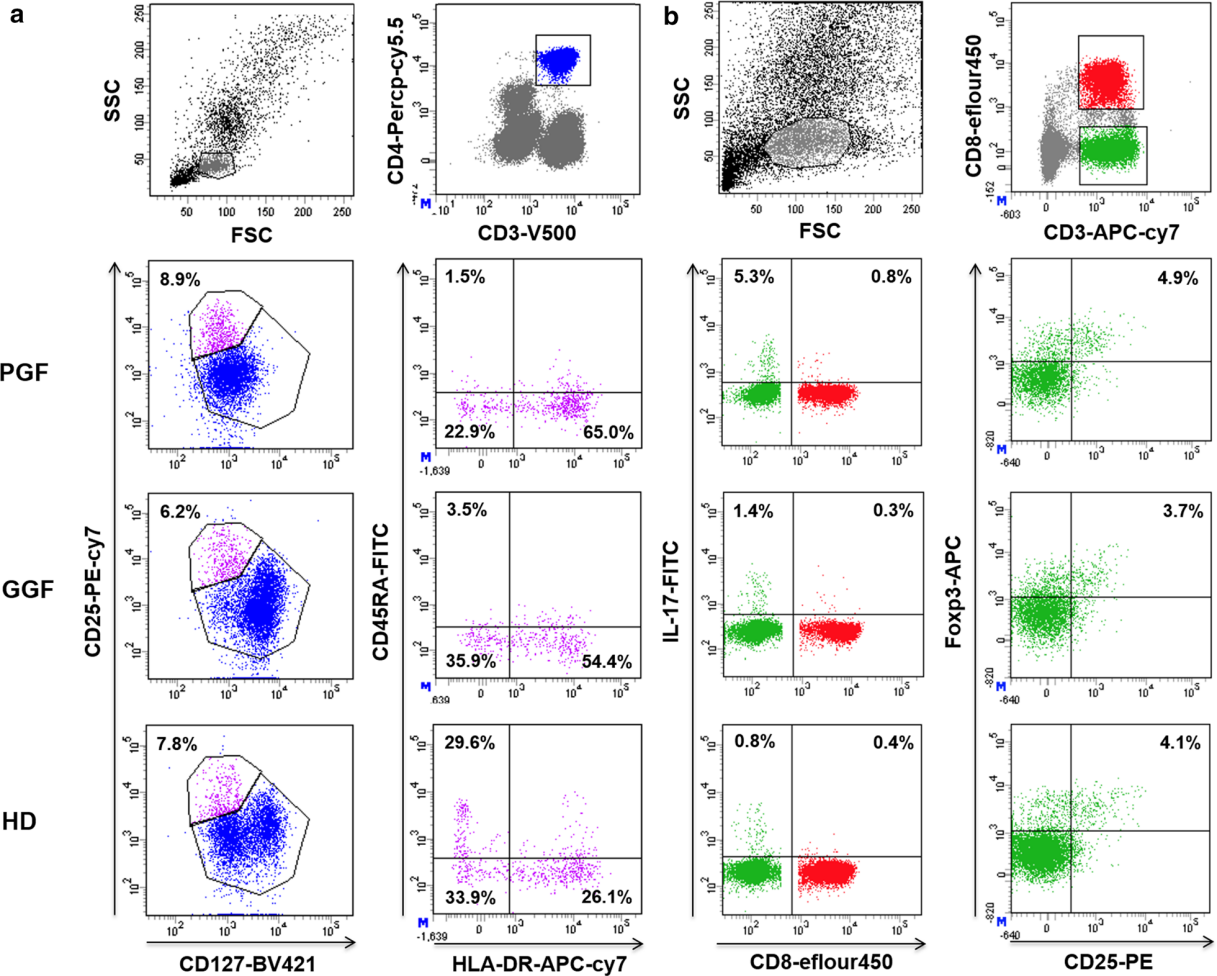
The lymphocyte population was initially defined using forward (FSC) and side scatter (SSC) gates. CD4^+^ T cells and CD8^+^ T cells were then gated based on CD4 and CD8 expression, respectively. **a** Representative flow cytometric analyses of CD25^+^CD127^−/dim^ Tregs and CD45RA^+^HLA-DR^−^ naïve Tregs from PGF patients, GGF patients, and HD. **b** Representative flow cytometric analyses of CD4^+^IL-17^+^ Th17, CD8^+^IL-17^+^ Tc17 cells, and CD25^+^Foxp3^+^ Tregs from PGF patients, GGF patients, and HD

Intracellular cytokine secretions were measured by flow cytometry after incubating the cells with phorbol myristate acetate (100 ng/mL; Sigma, St. Louis, MO, USA) and ionomycin (2 μg/mL Sigma, St. Louis, MO, USA) for 4 h to stimulate maximal IFN-γ, IL-4 and IL-17 production. GolgiStop (0.7 μL/mL) was added to the samples during this 4-h incubation to sequester proteins in the cytoplasm. The monoclonal antibodies APC-H7-conjugated anti-CD3, PE-conjugated anti-IL-4 (BD Biosciences), eFluor450-conjugated anti-CD8, PE-Cy7-conjugated anti-CD25, PerCP-Cy5.5-conjugated anti-IFN-γ, eFluor660-conjugated anti-Foxp3 and Alexa Fluor 488-conjugated anti-IL-17A (eBioscience, San Diego, CA, USA) were used to distinguish cell surface markers and intracellular cytokines. The typical gating strategies for the different T cell subsets in the BM of PGF, GGF, and HD are shown in Fig. 1. CD3^+^CD8^−^IFN-γ^+^, CD3^+^CD8^−^IL-4^+^, CD3^+^CD8^−^IL-17A^+^, and CD3^+^CD8^−^CD25^+^Foxp3^+^ cells were defined as Th1, Th2, Th17 cells and Tregs, respectively. Tc1, Tc2 and Tc17 cells were identified as CD3^+^CD8^+^IFN-γ^+^ CD3^+^CD8^+^IL-4^+^, and CD3^+^CD8^+^IL-17A^+^, respectively. For Treg subsets analyses, CD4^+^CD25^+^CD127^−/dim^CD45RA^+^HLA-DR^−^, CD4^+^CD25^+^CD127^−/dim^CD45RA^−^HLA-DR^−^, and CD4^+^CD25^+^CD127^−/dim^CD45RA^−^HLA-DR^+^ cells were further defined as naïve Tregs, memory Tregs and active Tregs [36], respectively. For the definition of other Treg subsets, CD45RA^+^CD25^dim^, CD45RA^−^CD25^high^, and CD45RA^−^CD25^dim^ represented resting Tregs, active Tregs and nonsuppressive Tregs [37], respectively. Percentages of cytokine-producing cells were calculated using overall CD4^+^ or CD8^+^ T cell subpopulations rather than total T cell populations. Type 1/type 2 ratios were calculated using the percentage of IFN-γ-producing cells divided by the percentage of IL-4-producing cells, whereas the ratio of Th17 cells/Tregs was calculated as the percentage of IL-17-producing cells divided by the percentage of CD25^+^Foxp3^+^ T cells.

### Statistical analysis

Median values and ranges are reported for continuous variables, and proportions are reported for categorical variables. Characteristics of the patients in the PGF and GGF groups were compared using the Chi square test for categorical variables and the Mann–Whitney *U* test for continuous variables. Analyses were performed using GraphPad Prism 6.0 (GraphPad Software, La Jolla, CA), and *P* values <0.05 were considered statistically significant.

## Results

### Patient characteristics

This prospective nested case–control study enrolled 20 patients with PGF, 40 matched patients with GGF after allo-HSCT and 20 HD. As shown in Table 1, PGF and GGF patients had their BM microenvironment tested at a matched median time point after allo-HSCT (102 days vs. 92.5 days, *P* = 0.14) to minimize the potential influence of the length of time after allo-HSCT. Additionally, polymerase chain reaction DNA fingerprinting of the STRs of the recipient peripheral blood showed complete donor chimerism in all patients.

**Table 1:** Characteristics of allo-HSCT patients with PGF and GGF

Characteristics	PGF* (n = 20)	GGF* (n = 40)	*P* value**
BM evaluated time (post-HSCT days)	102 (53–152)	92.5 (24–561)	0.14
Blood cell count			
Median WBC (×10^9^/L) (range)	1.1 (0.3–2.7)	5.01 (1.93–9.83)	<0.0001
Median ANC (×10^9^/L) (range)	0.7 (0.1–1.8)	2.62 (0.84–7.1)	0.0007
Median Hb (g/L) (range)	83 (68–104)	114.5 (85–165)	<0.0001
Median PLT (×10^9^/L) (range)	29 (4–53)	149.5 (31–266)	<0.0001
Age at HSCT (years, median, range)	33.5 (11–62)	26 (7–51)	0.10
Gender (male/female)	15/5	24/16	0.39
Underlying disease			0.78
AML	6	14	
ALL	9	16	
CML	0	2	
MDS	3	4	
sAA	2	4	
Status at HSCT			0.54
Standard-risk	4	12	
High-risk	16	24	
Source of stem cell			0.99
BM and G-PB	19	38	
G-PB	1	2	
Transplanted total nucleated cell dose (×10^8^/kg, median, range)	8.08 (6.01–14.49)	7.615 (5.22–13.81)	0.68
Transplanted CD34^+^ cell dose (×10^8^/kg, median, dose)	2.29 (1.18–0.5.28)	2.49 (0.85–6)	0.09
Donor match			0.34
HLA-identical unrelated donor	1	2	
HLA-identical sibling donor	3	12	
HLA-partially matched related	16	26	
Sex mismatch			0.99
Female to male	5	9	
Female to female	2	2	
Male to female	4	13	
Male to male	9	16	
ABO mismatch			0.47
No	12	26	
Minor	3	6	
Major	5	8	
Pre-HSCT cycles of chemotherapy	4 (0–6)	3.5 (0–11)	0.86
Conditioning			0.34
BU/CY	3	12	
BU/CY + ATG	17	28	
History of aGvHD	13	21	0.77
History of CMV reactivation	17	24	0.08

*allo-HSCT* allogeneic haematopoietic stem cell transplantation, *PGF* poor graft function, *GGF* good graft function, *AML* acute myelogenous leukemia, *ALL* acute lymphocytic leukemia, *CML* chronic myelogenous leukemia, *MDS* myelodysplastic syndrome, *sAA* sever aplastic anemia, *HLA* human leukocyte antigen, *BU* busulfan, *CY* cyclophosphamide; *ATG* anti-human thymus globulin; *aGvHD* acute graft-versus-host disease, *CMV* cytomegalovirus

* Group matching criteria included age at HSCT (±1 years), pre-HSCT cycles of chemotherapy (±1 cycle), disease status at HSCT and BM microenvironment evaluated time after HSCT (±5 days). For each PGF case, two GGF control was randomly selected from the same cohort at which the PGF occurred (“risk-set sampling”)

** The continuous variables were compared using the Mann–Whitney U test, and the differences in frequency between the 2 groups were compared using the Chi square test. The criterion for statistical significance was *P* < 0.05

The demographic and clinical characteristics of PGF and GGF patients, including age, gender, underlying disease, disease status pre-HSCT, median time from diagnosis to HSCT, source of stem cells, transplanted total nucleated cell dose, CD34^+^ cell dose, donor HLA match, sex/ABO mismatch, pre-HSCT cycles of chemotherapy, preparative regimens, GvHD prophylaxis and history of GvHD and CMV status, were comparable (Table 1).

### Blood and BM cellularity

**Fig. 2:**
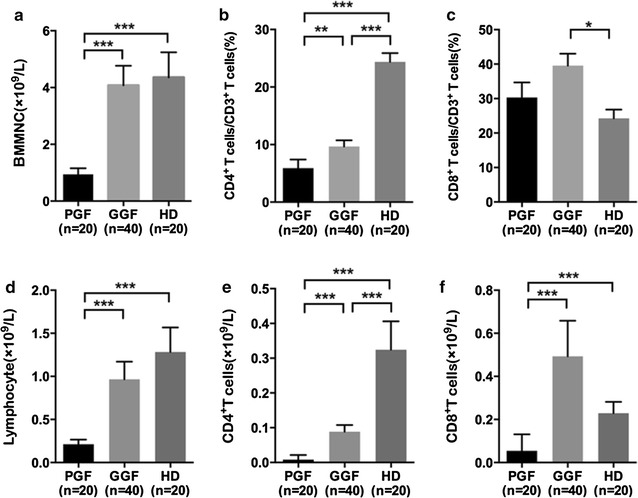
The absolute numbers of BMMNCs (**a**), lymphocytes (**d**), CD4^+^ T cells (**e**) and CD8^+^ T cells (**f**). The percentages of CD4^+^ T cells (**b**) and CD8^+^ T cells (**c**). Statistical analyses were performed using the Mann–Whitney *U* test. *P values < 0.05; **P values < 0.005; ***P values < 0.0001

The hemograms of PGF patients showed significant pancytopenia compared with those of GGF patients. The median WBC (1.1 × 10^9^/L vs. 5.0 × 10^9^/L, *P* < 0.0001), ANC (0.7 × 10^9^/L vs. 2.6 × 10^9^/L, *P* = 0.0007), Hb (83 g/L vs. 114.5 g/L, *P* < 0.0001) and PLT (29 × 10^9^/L vs. 149.5 × 10^9^/L, *P* < 0.0001) levels in the PGF group were dramatically lower than those of the GGF group when the BM was evaluated (Table 1). The BMMNCs in the PGF group were significantly reduced compared with those in the GGF group (Fig. 2; 0.8 × 10^9^/L vs. 2.6 × 10^9^/L, *P* < 0.0001).

### Lymphocyte subsets in BMMNCs

The median percentages and absolute quantities of T lymphocyte subpopulations in BMMNCs from PGF patients, GGF patients, and HD are provided in Additional file 1: Table S1. Conspicuous lymphopenia was exhibited in the PGF group. Lymphocyte percentages in the PGF and GGF group were slightly lower than those in the HD group. Thus, the observed lymphopenia was primarily caused by an overall reduction in the absolute values of T lymphocyte subgroups in BMMNCs, and the subtle decrease in lymphocyte percentage may have had an influence as well. As shown in Additional file 1: Table S1, the median value of absolute counts of lymphocytes (0.1 × 10^9^/L vs. 0.5 × 10^9^/L, *P* < 0.0001), CD4^+^ T cells (0.003 × 10^9^/L vs. 0.04 × 10^9^/L, *P* < 0.0001), and CD8^+^ T cells (0.01 × 10^9^/L vs. 0.2 × 10^9^/L, *P* < 0.0001) were significantly decreased in PGF patients compared with those in GGF patients. The median percentages of CD8^+^ T cells (30.6 vs. 42.6%, *P* = 0.13) between the PGF and GGF groups showed no significant difference, whereas the median percentage of CD4^+^ T cells (3.4 vs. 8.4%, *P* = 0.004) was significantly decreased in PGF patients.

In terms of the CD4^+^ and CD8^+^ subsets, the state of activation was evaluated based on the surface expression of HLA-DR. As shown in Additional file 1: Table S1, the percentage of activated CD8^+^ T cells and active CD4^+^ T cells in the BM immune microenvironment was significantly higher in PGF patients than in GGF patients. With the exception of activated CD4^+^ T cells and CD8^+^ T cells, the absolute quantities of cell subsets were significantly lower among PGF patients than among HD. Compared with HD, PGF and GGF patients showed lower levels of naïve phenotypes for both CD4^+^ T cells and CD8^+^ T cells, but higher levels of effector CD8^+^ T cells. In addition, the percentages of effector memory CD4^+^ T cells and effector memory CD8^+^ T cells were elevated PGF and GGF patients compared with HD.

### Increased expression of Th17, Tc17, Th1, and Tc1 cells in the BM of PGF patients

**Fig. 3:**
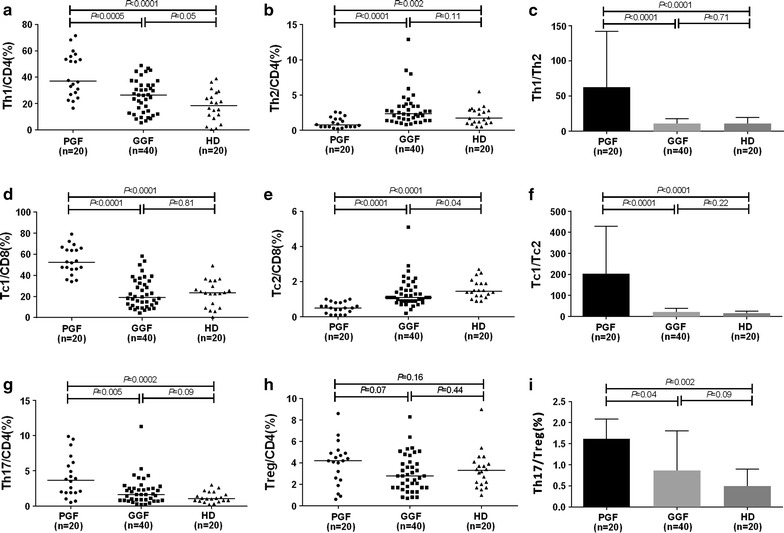
The percentages of the Th1 cell (**a**), Th2 cell (**b**), Th17 cell (**g**) and Treg (**h**) subsets among CD4^+^ T cells and the percentages of the Tc1 (**d**), and Tc2 (**e**) cell subsets among CD8^+^ T cells. Th1 cell/Th2 cell (**c**), Tc1 cell/Tc2 cell (**f**) and Th17 cell/Treg (**i**) ratios. Statistical analyses were performed using the Mann–Whitney *U* test

We first analyzed the frequency of Th17 cells, Tc17 cells and Tregs in PGF patients, GGF patients and HD. A representative dot plot of the percentages of Th17 cells, Tc17 cells and Tregs in representative PGF patients, GGF patients and HD is shown in Fig. 1. The percentages of Th1, Th2 and Th17 cells among CD4^+^ T cells and the percentages of Tc1, Tc2 and Tc17 cells among CD8^+^ T cells are shown in Fig. 3. The percentages of Th1 (37 vs. 26.4%, *P* = 0.0005) and Tc1 (52.4 vs. 19%, *P* < 0.0001) cells were significantly higher in PGF patients than in GGF patients, whereas the percentages of Th2 (0.8 vs. 2.4%, *P* < 0.0001) and Tc2 (0.5 vs. 1.1%, *P* < 0.0001) cells were markedly lower in PGF group than in GGF group. The median percentages of Th1 (26.4 vs. 18.5%, *P* = 0.05), Tc1 (19 vs. 23.6%, *P* = 0.81), and Th2 cells (2.4 vs. 1.8%, *P* = 0.11) showed no significant differences between GGF group and HD group, whereas the percentage of Tc2 cells (1.1 vs. 1.5%, *P* = 0.04) in GGF group was lower than that in HD group.

The type 1/type 2 immune response ratio was calculated using the Th1 cell/Th2 cell and Tc1 cell/Tc2 cell ratios. PGF patients showed significantly greater median Th1 cell/Th2 cell ratio (31.6 vs. 10.8, *P* < 0.0001) and Tc1 cell/Tc2 cell ratio (108.8 vs. 18.4, *P* < 0.0001) than those for GGF patients, whereas similar Th1 cell/Th2 cell ratio (10.8 vs. 8, *P* = 0.71) and Tc1 cell/Tc2 cell ratio (18.4 vs. 14.8, *P* = 0.22) were found between GGF patients and HD.

We also evaluated the surface phenotypes of Tregs (Additional file 1: Figure S1). The percentages of CD45RA^−^HLA-DR^+^ active Tregs (61.2 vs. 51 vs. 18.0%, *P* < 0.05) were higher in PGF and GGF patients than HD, whereas the percentages of CD45RA^+^HLA-DR^−^ naïve Tregs were lower in PGF and GGF patients than in HD (1.1 vs. 2.9 vs. 24.9%, *P* < 0.05). Tregs were defined as CD4^+^CD25^+^Foxp3^+^ T cells after intracellular staining. The proportions of CD4^+^CD25^+^Foxp3^+^ Tregs among PGF patients, GGF patients and HD were comparable (Fig. 3, 4.5 vs. 2.8 vs. 3.3%, *P* > 0.05), and the percentages of CD4^+^CD25^+^CD127^−/dim^ Tregs showed no significant difference among patients with PGF, GGF and HD (Additional file 1: Figure S1). Compared to HD, the percentages of the resting Tregs were lower, whereas the percentages of active and nonsuppressive Tregs were higher in PGF and GGF patients (Additional file 1: Figure S1).

The proportions of Tregs among CD4^+^ T cells were comparable between PGF patients, GGF patients and HD. There was a significantly elevated percentage of Th17 cells in PGF patients compared with the percentage in GGF patients and HD (3.7 vs. 1.6 vs. 1.1%, *P* < 0.05). Consequently, a prominent increase in the Th17 cell/Treg ratio was observed in PGF group compared with the ratio in GGF and HD groups (1.0 vs. 0.6, *P* = 0.04), whereas similar Th17 cell/Treg ratio (0.6 vs. 0.4, *P* = 0.09) was observed between GGF group and HD group (Fig. 3).

## Discussion

In this nested case–control study, we demonstrated that PGF patients had a significantly higher proportion of stimulated CD4^+^ and CD8^+^ T cells that produced IL-17 (Th17 and Tc17), resulting in an IL-17-dominant response, whereas the proportions of Tregs among CD4^+^ T cells showed no significant differences between PGF and GGF patients. Therefore, a prominent increase in the Th17 cell/Treg ratio was observed in PGF patients compared with that of GGF patients. Additionally, we confirmed that both CD4^+^ and CD8^+^ T cells were polarized towards a type 1 immune response in PGF patients [17]. However, no significant different Th1 cell/Th2 cell, Tc1 cell/Tc2 cell and Th17 cell/Treg ratios were found between GGF patients and HD. These data suggest that dysregulated T cell responses may contribute to the occurrence of PGF post-allo-HSCT.

Rapid and persistent hematopoietic recovery plays a predominant role in successful allo-HSCT. Growing evidence suggests that BM-resident T cells may contribute to the formation of the perivascular BM immune microenvironment and participate in the regulation of hematopoiesis [13–16]. The Th1 cell/Th2 cell balance is well known to regulate the immune system under normal situations, whereas Th1 cell/Th2 cell imbalances have been reported in aplastic anemia (AA) and autoimmune diseases [38, 39]. The Th1 cell polarization of the immune response was reported in immune thrombocytopenia (ITP) in vitro [40] as well as in the BM immune microenvironment of ITP patients [25]. Moreover, our previous pilot study demonstrated an increased type 1 immune response in the BM immune microenvironment of PGF patients after allo-HSCT [17]. By enlarging the sample size, the current nested case–control study confirmed that both CD4^+^ and CD8^+^ T cells were polarized towards a type 1 immune response in PGF patients. Thus, it is plausible that deficits in the BM microenvironment due to aberrant immune responses by these CD4^+^ and CD8^+^ T cells may aggravate BM dysfunction.

Several groups have shown that Th17/Tc17 cells contribute to the pathogenesis of inflammation, autoimmune disease, tumors and hematopoiesis [41–43]. De Latour et al. [41]. reported an increased frequency of Th17 cells among BMMNCs and peripheral blood mononuclear cells in 41 patients with severe AA before any specific therapy at diagnosis, along with a reduction in Tregs. Evidence from a murine study showed that Th17 cells impact cell fate and function via the mTOR signaling pathway and metabolic processes [44]. In the current study, the proportions of Th17 and Tc17 cells were found to be significantly increased in the BM microenvironment of PGF patients compared with the proportions in GGF patients. These findings may indicate a very important role for Th17 and Tc17 cells in regulating HSCs recovery after allo-HSCT.

Tregs represent one-third of all CD4^+^ T cells in the BM microenvironment [27]. It has been shown in vitro that Treg defects may contribute to impaired hematopoiesis mediated by effector T cells in acquired AA, and increased autoreactive T cells may promote the development of AA [45, 46]. In mouse transplantation models, allogeneic HSCs have been reported to protect against allorejection by host Tregs, which raises the possibility that Tregs contribute to maintaining immune-privileged sites [27]. However, no significant differences were found in the percentages of Tregs between the PGF and GGF groups, whereas a prominent increase in the Th17 cell/Treg ratio was observed in PGF patients compared with GGF patients.

Aberrant T cell responses, including both CD4^+^ and CD8^+^ T cells, polarized towards a type 1 immune response as well as a Th17 cell/Treg imbalance were found in the BM immune microenvironment of PGF patients. Considering the crucial role of the BM immune microenvironment in supporting hematopoiesis [13–16], we hypothesize that a dysregulated BM immune microenvironment may hamper the hematopoietic reconstitution of successfully engrafted donor HSCs, ultimately leading to the occurrence of PGF post-allotransplant.

We are aware, however, that additional functional studies are required to illuminate the direct interactions between the dysregulated immune cells and HSCs or other cellular elements of the BM microenvironment, as well as the molecular mechanisms underlying this phenomenon. Moreover, although the RBC and PLT transfused to the patients post-HSCT were gamma irradiated, future studies are needed to elucidate whether the increased number of CD8^+^ effector cells in PGF patients are due to responses resulting from the transfusion.

## Conclusions

In conclusion, the present study revealed that imbalances in the Th1 cell/Th2 cell, Tc1 cell/Tc2 cell, and Th17 cell/Treg ratios in the BM immune microenvironment of PGF patients compared with GGF patients. Although requiring further validation, these data suggest that aberrant T cell responses in the BM immune microenvironment appear to be involved in the occurrence of PGF. Therefore, it would be of value to investigate whether potential immunotherapy targeting aberrant T cell responses could improve hematopoietic reconstitution by correcting the impaired BM immune microenvironment in PGF patients post-allotransplant in the future.

## Additional file

**Additional file 1.** Distribution of the T lymphocyte subsets in bone marrow of patients with PGF or GGF and HD.

## Abbreviations

PGF: poor graft function
allo-HSCT: allogeneic hematopoietic stem cell transplantation
BM: bone marrow
Tregs: regulatory T cells
GGF: good graft function
HD: healthy donors
EPCs: endothelial progenitor cells
IL: interleukin
GvHD: graft-versus-host disease
CMV: cytomegalovirus
rhG-CSF: recombinant human granulocyte colony-stimulating factor
ANC: absolute neutrophil cell
PLT: platelet
Hb: hemoglobin
STRs: short tandem repeats
BMMNCs: bone marrow mononuclear cells
WBCs: white blood cells
AA: aplastic anemia
ITP: immune thrombocytopenia
