# Supplementary material for: Aberrant T cell responses in the bone marrow microenvironment of patients with poor graft function after allogeneic hematopoietic stem cell transplantation
Source: J Transl Med. 2017 Mar 14;15:57. doi: 10.1186/s12967-017-1159-y (PMC5351211; doi:10.1186/s12967-017-1159-y)

**Supplement Table 1. Distribution of the T lymphocyte subsets in bone marrow of patients with PGF or GGF and HD**

|  | **PGF (n=20)** | **GGF (n=40)** | **HD (n=20)** | ***P*-value^1^** | ***P*-value^2^** | ***P*-value^3^** | ***P*-value^4^** |
| --- | --- | --- | --- | --- | --- | --- | --- |
| BMMNCs (×10^9^/L) | 0.8(0.07-3) | 2.6(0.3-21) | 3.5(0.5-11) | <0.0001 | 0.0001 | 0.0004 | 1.00 |
| Lymphocytes |  |  |  |  |  |  |  |
| Percentage of BMMNCs (%) | 19.7 (3.5-67.2) | 25.9(3.9-67.5) | 32 (8.7-825) | <0.0001 | 0.99 | 0.11 | 0.16 |
| Absolute value (×10^9^/L) | 0.1(0.009-0.7) | 0.5 (0.07-6.8) | 0.87 (0.1-3.4) | <0.0001 | <0.0001 | 0.0001 | 0.57 |
| CD4^+^ T cells |  |  |  |  |  |  |  |
| Percentage of lymphocytes (%) | 3.4(0.1-24.4) | 8.4(1.1-29.3) | 23.3 (14.9-36.1) | <0.0001 | 0.004 | <0.0001 | <0.0001 |
| Absolute value (×10^9^/L) | 0.003 (0-0.05) | 0.04 (0.001-0.55) | 0.21(0.02-0.92) | <0.0001 | <0.0001 | <0.0001 | 0.02 |
| CD4^+^HLA-DR^+^ T cells |  |  |  |  |  |  |  |
| Percentage of CD4^+^ T cell (%) | 48.6 (5.4- 77.6) | 22.5 (3.6-77.2) | 7.9 (3.2-22.3) | <0.0001 | 0.02 | <0.0001 | 0.01 |
| Absolute value (×10^9^/L) | 0.001 (0-0.02) | 0.009 (0-0.35) | 0.02 (0.001-0.12) | 0.0001 | 0.001 | 0.0004 | 0.82 |
| Effector CD4^+^ T cells |  |  |  |  |  |  |  |
| Percentage of CD4^+^ T cells (%) | 1.7 (0-10) | 1.6 (0.1-21.3) | 1.1 (0-9.6) | 0.93 | 0.99 | 0.99 | 0.99 |
| Absolute value (×10^9^/L) | 0 (0-0.002) | 0.001 (0-0.06) | 0.001 (0-0.04) | <0.0001 | 0.0007 | 0.0002 | 0.68 |
| Naïve CD4^+^ T cells |  |  |  |  |  |  |  |
| Percentage of CD4^+^ T cells (%) | 3.6 (0.2-40.9) | 3 (0.1-52) | 35.9 (1.7-76.2) | <0.0001 | 0.99 | <0.0001 | <0.0001 |
| Absolute value (×10^9^/L) | 0 (0-0.002) | 0.001 (0-0.15) | 0.08 (0-0.57) | <0.0001 | 0.004 | <0.0001 | 0.0005 |
| Effector memory CD4^+^ T cells |  |  |  |  |  |  |  |
| Percentage of CD4^+^ T cells (%) | 67.5 (14.4-84.6) | 70.3 (14.9-94.8) | 26.3(8.7-74.6) | <0.0001 | 0.99 | 0.0008 | <0.0001 |
| Absolute value (×10^9^/L) | 0.001 (0-0.04) | 0.02 (0.001-0.34) | 0.05(0.004-0.69) | <0.0001 | <0.0001 | <0.0001 | 0.68 |
| Central memory CD4^+^ T cells |  |  |  |  |  |  |  |
| Percentage of CD4^+^ T cells (%) | 28.95 (11.9-49) | 20.4 (2-45.8) | 28.6 (11.2-48.5) | 0.07 | 0.38 | 0.99 | 0.11 |
| Absolute value (×10^9^/L) | 0.001 (0-0.006) | 0.006 (0-0.18) | 0.08(0.004-0.18) | <0.0001 | 0.0002 | <0.0001 | 0.006 |
| CD8^+^ T cells |  |  |  |  |  |  |  |
| Percentage of lymphocytes (%) | 30.6 (0.6-66.8) | 42.6 (6.1-84.4) | 22.95(14.1-55.2) | 0.04 | 0.13 | 0.99 | 0.05 |
| Absolute value (×10^9^/L) | 0.01 (0.001-0.30) | 0.2 (0.007-5.7) | 0.15 (0.04-0.67) | <0.0001 | <0.0001 | 0.004 | 0.99 |
| CD8^+^HLA-DR^+^ T cells |  |  |  |  |  |  |  |
| Percentage of CD8^+^ T cells (%) | 64.9 (34.3-84.8) | 26.7 (6.3-82.8) | 18.9 (8.5-39.3) | <0.0001 | <0.0001 | <0.0001 | 0.97 |
| Absolute value (×10^9^/L) | 0.008 (0-0.11) | 0.04 (0-2.56) | 0.03 (0.006-0.11) | 0.05 | 0.05 | 0.92 | 0.89 |
| Effector CD8^+^ T cells |  |  |  |  |  |  |  |
| Percentage of CD8^+^ T cells (%) | 48.4 (3.3-73.4) | 41.3 (6.3-72.8) | 33.5 (7.9-66.6) | 0.08 | 0.31 | 0.08 | 0.99 |
| Absolute value (×10^9^/L) | 0.003 (0-0.08) | 0.02 (0-1.08) | 0.008 (0.002-0.07) | 0.04 | 0.04 | 0.99 | 0.55 |
| Naïve CD8^+^ T cells |  |  |  |  |  |  |  |
| Percentage of CD8^+^ T cells (%) | 3.55 (0.4-31.0) | 2.95 (0.4-46.6) | 29.05 (1-57.9) | <0.0001 | 0.99 | <0.0001 | <0.0001 |
| Absolute value (×10^9^/L) | 0 (0-0.009) | 0.006 (0-0.33) | 0.05 (0-0.38) | <0.0001 | 0.0001 | <0.0001 | 0.06 |
| Effector memory CD8^+^ T cells |  |  |  |  |  |  |  |
| Percentage of CD8^+^ T cells (%) | 40.95(14-95) | 48.75 (13.2-85.3) | 33.85 (8.8-51.1) | 0.002 | 0.99 | 0.07 | 0.001 |
| Absolute value (×10^9^/L) | 0.007 (0-0.18) | 0.09 (0.003-2.38) | 0.05 (0.02-0.22) | <0.0001 | <0.0001 | 0.10 | 0.32 |
| Central memory CD8^+^ T cells |  |  |  |  |  |  |  |
| Percentage of CD8^+^ T cells (%) | 2.2 (0.5-11) | 2.1 (0.3-21.4) | 3.6 (0.8-9.3) | 0.08 | 0.99 | 0.12 | 0.17 |
| Absolute value (×10^9^/L) | 0 (0-0.004) | 0.005 (0-0.16) | 0.007 (0-0.04) | <0.0001 | <0.0001 | <0.0001 | 0.99 |

Data are expressed as median value (range).

Statistical analyses among the 3 groups were performed using 1-way ANOVA, and the Dunn’s multiple comparison test was used for multiple comparisons between 2 groups. Analyses were performed using GraphPad Prism 6.0 (GraphPad Software, La Jolla, CA), and *P*-values <0.05 were considered statistically significant.

***P*-value^1^:** The *P*-value of comparison among PGF, GGF and HD groups.

***P*-value^2^:** The *P*-value of comparison between PGF group and GGF group.

***P*-value^3^:** The *P*-value of comparison between PGF group and HD group.

***P*-value^4^:** The *P*-value of comparison between GGF group and HD group.

**Abbreviations:** HD indicates healthy donor; BMMNCs, bone marrow mononuclear cells.

**Supplement Figure 1**. The percentages of CD25^+^CD127^dim/-^ Tregs **(A)** and Treg subsets **(B-C)**. Statistical analyses were performed using the Mann–Whitney *U* test.


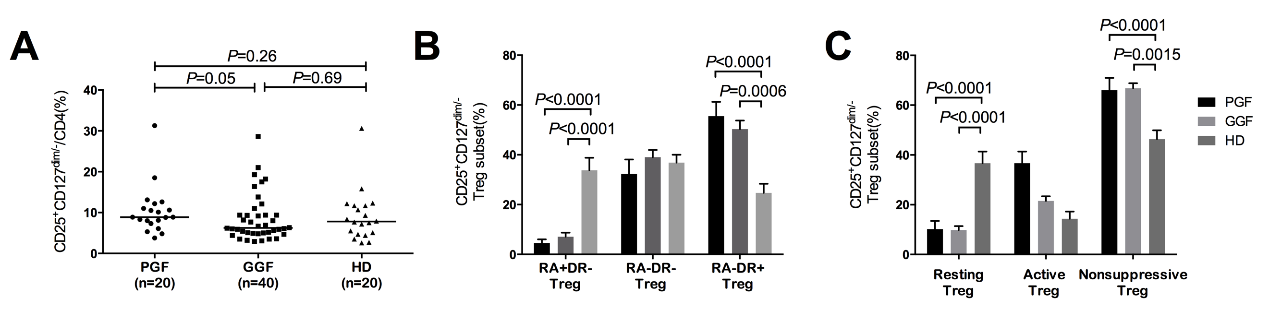

Supplement: Supplementary file 1 — Additional file 1. Distribution of the T lymphocyte subsets in bone marrow of patients with PGF or GGF and HD. [file 12967_2017_1159_MOESM1_ESM.docx]
